# De novo genome assembly of endophytic fungus *Nemania diffusa* strain YAFEF818, isolated from *Artemisia argyi*

**DOI:** 10.1128/mra.00360-24

**Published:** 2024-07-08

**Authors:** Jiaojun Yu, Xiaolong Yuan, Yan Kui, Yunqin Li, Wei Bi, Yi Wang

**Affiliations:** 1 Hubei Key Laboratory of Economic Forest Germplasm Improvement and Resources Comprehensive Utilization, Huanggang Normal University, Huanggang, Hubei, China; 2 Yunnan Key Laboratory of Biodiversity of Gaoligong Mountain, Yunnan Academy of Forestry & Grassland, Kunming, Yunnan, China; 3 Resource Protection Station, Chengjiang Forestry and Grassland Bureau, Chengjiang, China; Rochester Institute of Technology, Rochester, New York, USA

**Keywords:** *Nemania diffusa*, genome, *Artemisia argyi*

## Abstract

Here, we report a draft genome sequence of endophytic fungus *Nemania diffusa* YAFEF818, isolated from *Artemisia argyi*. Oxford Nanopore Technologies PromethION and Illumina NovaSeq sequence reads were assembled using NECAT and polished using pilon to yield a 55.63 Mb genome.

## ANNOUNCEMENT

Endophytic fungi live in healthy plant tissues without causing disease symptoms in their host plants (1). They can produce a large number of active compounds including xanthones, terpenoids, isocoumarins, and enniatines (2, 3). *Nemania diffusa* can produce a series of enzymes such as phenoloxidases, peroxidase, which are able to neutralize fungitoxic compounds (4). In this study, we report a new genome of endophytic fungus *N. diffusa* strain YAFEF818 from the roots of *A. argyi*.

*N. diffusa* strain YAFEF818 was isolated from the roots of *A. argyi*. The isolation process was as follows: the roots were collected from Jinggu, Yunnan Province, China (23°50′12″ N, 100°02′34″ E) in 2019 and soaked in tap water for 24 h to clean them. They were then rinsed with sterile water for 5 min. The roots were ground in a ball mill (GT100; GRINDER, Beijing, China) at 180 rpm and 25°C, and sterile water was added to obtain a slurry. Finally, the root slurry was diluted and spread onto solid media PDA (Potato Dextrose Agar) with 100 µg/mL ampicillin, kanamycin, and streptomycin. Single clone was transferred to fresh PDA media after 5 days of incubation at 28°C. A single colony (strain YAFEF818) was identified as *N. diffusa* by comparing it with the known ITS region of *N. diffusa*. The ITS sequence was deposited in GenBank (accession number OR481915).

The mycelia of *N. diffusa* were harvested for DNA extraction after cultivation at 28°C for 10 days on a rotary shaker at 150 rpm. Genomic DNA was extracted using a DNeasy Mini kit (Qiagen, USA) following the manufacturer’s guidelines. The library preparation for the Illumina NovaSeq platform with insert sizes of 400 bp, and a 2 × 150 bp paired-end configuration was performed using the TruSeq DNA Sample Prep Kit (New England Biolabs, USA) following the Illumina TruSeq DNA sample preparation guide. And for the Nanopore PromethION platform was executed according to the ligation sequencing SQK-LSK110 kit (ONT, UK) according to the standard protocol. No shearing or size selection was performed during library preparation. The library was sequenced using a single FLO-MIN106 (R9.4.1) flow cell for 72 h on a PromethION sequencer. Reads were base called using Guppy (version 0.3.0), resulting in 944,396 reads with a N50 of 17,248 bp. In all, 981,310,309 bp (coverage, 118×) of data passed quality control. The filtered Nanopore reads were assembled using NECAT (5). Default parameters were used for all software unless otherwise specified. Racon (version:1.4.11) tool was used to perform two rounds of error correction on the splicing result based on the third-generation sequencing data (6). For Illumina short reads, quality control was performed with AdapterRemoval version 2 (7). Pilon package version 1.23 (8) was used to correct the base errors of the assembled contigs by using the Illumina short reads. The assembly resulted in 19 scaffolds with a total assembly length of 55,636,603 bp (N50, 4,970,375 bp; N90, 3,935,343 bp), a GC content of 39.9% (Table 1). The genome completeness was assessed using BUSCO v3.0.2 (9), showing a completeness score of 98.80%.

**TABLE 1:** Genome sequencing statistics

Parameter	Value
Illumina sequencing	
Raw reads	
Reads number	44,555,054
Total bases (bp)	6,727,813,154
Nanopore sequencing	
Total sequence number	944,396
Total sequence length	6,614,374,721
Genome assembly	
Total base	55,636,603
Total scaffolds	19
Max sequence length	7,158,893 bp
N50	4,970,375 bp
N90	3,935,343 bp
GC_content (%)	39.9
Genome assembly assessment	
Complete BUSCOs	98.80%
Complete and single-copy BUSCOs	98.80%
Complete and duplicated BUSCOs	0.00%

## Data Availability

This study project is available under NCBI BioProject accession number PRJNA1091577 and BioSample accession number SAMN40599169 and SRA accession number SRR29234302. The draft genome assembly of *N. diffusa* is available under GenBank accession number JBBPUQ000000000.
